# Body painting to promote self-active learning of hand anatomy for preclinical medical students

**DOI:** 10.3402/meo.v21.30833

**Published:** 2016-03-02

**Authors:** Pitchanee Jariyapong, Chuchard Punsawad, Suchirat Bunratsami, Paranyu Kongthong

**Affiliations:** School of Medicine, Walailak University, Nakhonsrithammarat, Thailand

**Keywords:** body painting, living anatomy, medical education, learning and teaching

## Abstract

**Background:**

The purpose of this study was to use the body painting method to teach hand anatomy to a group of preclinical medical students.

**Methods:**

Students reviewed hand anatomy using the traditional method and body painting exercise. Feedback and retention of the anatomy-related information were examined by a questionnaire and multiple-choice questions, respectively, immediately and 1 month after the painting exercise.

**Results:**

Students agreed that the exercise was advantageous and helped facilitate self-active learning after in-class anatomy lessons. While there was no significant difference in knowledge retention between the control and experimental groups, the students appreciated the exercise in which they applied body paint to the human body to learn anatomy.

**Conclusion:**

The body painting was an efficient tool for aiding the interactive learning of medical students and increasing the understanding of gross anatomy.

In medical schools, gross anatomy is the branch of biology in which the human body is studied using a cadaver and often requires a great deal of effort for students to memorize the various structures. However, medical students generally have no chance to directly transfer their knowledge from the laboratory to a living body (1) and it is not enough for a clinician to evaluate structure or organs of patients in the clinical practice (2). The novel learning and teaching methodology of living anatomy reveals the bodily structures of a living human. It is currently gaining importance in modern anatomy education and has been considered as a replacement for cadaver-based anatomy study (3). Through the three main modalities of living anatomy – surface anatomy, medical imaging, and surgical procedures – students are able to engage in self-directed learning (SDL), leading to positive anatomy education outcomes (4).

Body painting, a form of body art where the human skin is painted, has been reported as a useful adjunct to living anatomy, traditional anatomy courses, and clinical skills classes (5). To use this technique to teach anatomy, several structures – muscles, vessels, bones, nerves, and internal organs – are painted onto an actual living human body, allowing for easy palpation and examination. Medical students have been reported as being engaged during the body painting session and having fun. This also presents the opportunity to accommodate students with a variety of learning styles (6), aiding their retention of anatomical knowledge (7). In the present study, we used the body painting method to teach hand anatomy to a group of students. We selected this structure because it is easy to observe the distal end of each arm and contains no gender-specific organs. After the initial trial, the participating students later used body painting for learning about other anatomical regions of humans.

## Methods

### Student recruitment and color sensitivity test

First- and second-year preclinical medical students of Walailak University (*N*=96) were invited to participate in this project. All participating students signed individual consent forms, and ethical approval was provided by the Ethics Committee of Walailak University. Non-toxic water-washable body paints of various colors and brushes of different sizes were used for the body painting activity. Before beginning the session, the students performed tests to determine sensitivity to the paints used.

### Intervention and data collection

Students listened to an approximately 30-min lecture on anatomical terminology and the anatomy of the hand. After the lecture, the students were randomly divided into control and experimental groups, with 48 students each. In each group, students were further subdivided into six subgroups (*N*=8) for reviewing hand anatomy by different methods. The experimental subgroups had 20 min to paint only one structure – bones, muscles, nerve innervation and dermatome, or blood supply – on the hand of a partner and another 20 min to paint their own hand. These subgroups had an additional 20 min for discussion. Meanwhile, students in the control group studied hand anatomy using a handout and textbook for 1 h. The students’ short-term retention of the anatomy-related information was evaluated in both the experimental and control groups by a set of multiple-choice questions. Feedback was obtained from the experimental group by a structured questionnaire using a six-point Likert scale with agree/disagree categories (6). Long-term retention was evaluated in both groups using the same multiple-choice questions 1 month after the body painting activity.

## Results

### Body painting promoted active learning in anatomical classroom

**Fig. 1:**
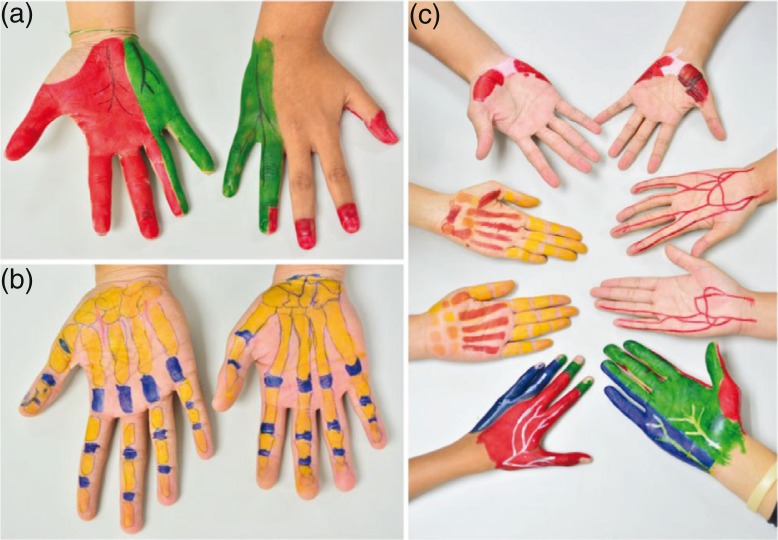
Body painting of dermatome (a) bone (b) and other related structures (c) in the hand.

**Fig. 2:**
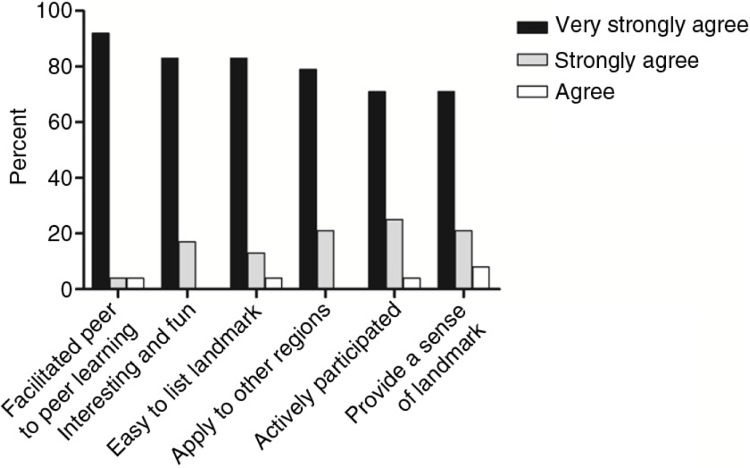
The percentages of agreement/disagreement with the closed questions for body painting exercise from the student.

Body painting is a helpful tool to illustrate the surface projection of desired structures and their relationship, particularly for the closely related structures in the hand (Fig. 1a–c). Students completed a feedback questionnaire and most stated they very strongly agreed that they actively participated in the body painting session (92%). In addition, 83% of students very strongly agreed that using body painting for learning hand anatomy is interesting and felt that the body painting exercise facilitated peer-to-peer learning. Approximately 79% of students very strongly agreed that the body painting method should be used to facilitate learning the anatomical structures of other regions of the body. Overall, 71% of students very strongly agreed that body painting helped them to easily list the structures of the hand and provided them with a sense of the landmarks in various structures. The percentages of agreement/disagreement with the closed questions for body painting exercise are shown in Fig. 2.

### Quantitative retention of knowledge

**Fig. 3:**
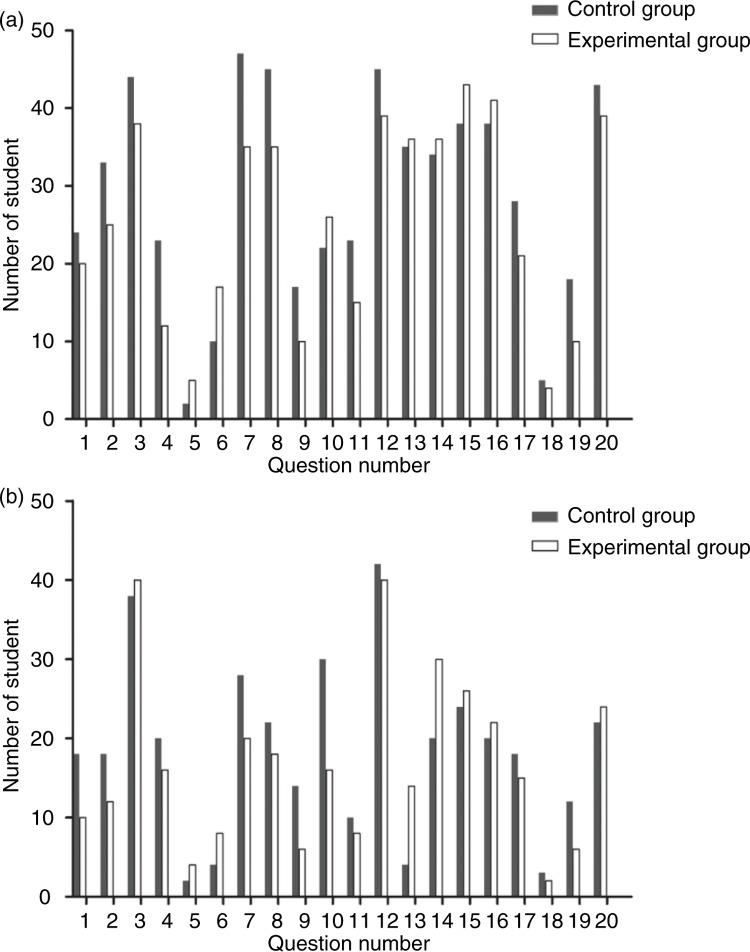
The number of students in the control and experimental groups who selected the correct answer immediately (a) and 1 month (b) following the body painting session.

Students’ short-term and long-term retention of knowledge was evaluated using a set of multiple-choice questions immediately after the body painting exercise and 1 month after the exercise, respectively. The results showed that the mean scores of the control and experimental groups immediately after the body painting session were 12.2±2.4 and 11.3±2.3, while the scores 1 month after the experiment were 8.0±2.5 and 7.4±2.8, respectively. There was no significant difference between the scores for each group both immediately after and at 1 month after the experiment. We further found that for questions involving the painted surface anatomy (e.g., questions 13–16), the number of students in the experimental group who selected the correct answer was higher than the number in the control group immediately following the body painting session (Fig. 3a). The same trend was found during evaluation 1 month after the experiment (Fig. 3b), indicating the impact of body painting on the retention of information.

## Discussion

In the twenty-first century, many students are becoming dependent on the Internet for receiving information and seeking additional resources. Therefore, the attitude toward and the teaching tools used for the process of teaching basic medical sciences, including anatomy in medical school, should be modified according to changes in behavior patterns. Innovations such as SDL, problem-based learning (PBL), and computer-assisted learning have been discussed as means to understand living and surface anatomy and to replace the dissection of human cadavers (3). In the present study, we report body painting as a teaching methodology to study the anatomy of the hand and promote self-active learning.

The shift of traditional teaching methods to an integrated teaching approach using body painting has long been reported (8, 9). Apart from relaxation of students from intensive reading of textbooks, visual stimuli with tactile painting facilitate recall of knowledge (10). In this study, despite no significant difference between the retention of knowledge between the two groups during evaluation immediately following the experiment (short-term retention) or 1 month after (long-term retention), the experimental group was allowed a shorter time (20 min) for study compared to the control group (1 h). The body painting exercise thus considerably promotes active learning whereby students search for tools to achieve understanding of the subject rather than simply repeating what they have learned in anatomy classes (11). Students are encouraged to be active learners and change their position from being a listener to being a didactic teacher, which is at the bottom of Dales’ cone of experience (12) and can result in up to 90% knowledge retention.

Here, our results correspond with other reports that body painting is a useful tool for teaching anatomy (6, 13). With a decline in the number of cadavers available for medical education, body painting may be a realistic option for learning surface anatomy. Compared with other living anatomy modalities, the costs of body painting are inexpensive and the enjoyable activity can be concurrently performed with large class sizes.
